# Prevention of Dental Erosion Caused by Fermented Milk: An In-vitro Study

**DOI:** 10.3290/j.ohpd.b3556031

**Published:** 2022-11-08

**Authors:** Kyung-hee Kim, Choong-Ho Choi, Ki-Ho Chung

**Affiliations:** a Assistant Professor, Department of Dental Hygiene, Hanyeong University, Yeosu, Korea. Data acquisition, analysis and interpretation; drafted and critically revised the manuscript, gave final approval and agreed to be accountable for all aspects of the work.; b Professor, Department of Preventive and Public Health Dentistry, Chonnam National University School of Dentistry, Gwangju, Korea. Data acquisition, analysis and interpretation; drafted and critically revised the manuscript, gave final approval and agreed to be accountable for all aspects of the work; c Professor, Department of Preventive and Public Health Dentistry, Chonnam National University School of Dentistry, Gwangju, Korea. Conception, design, and interpretation, critically revised the manuscript, gave final approval and agreed to be accountable for all aspects of the work.

**Keywords:** calcium, dental erosion, fermented milk, fluoride

## Abstract

**Purpose:**

This study aimed to assess the efficacy of three techniques for preventing dental erosion and thereby identify effective methods. The three techniques were: 1) adding calcium to fermented milk (2% Ca and 0.5% Ca); 2) topical application of fluoride to the teeth before exposure to fermented milk (acidulated phosphate fluoride [APF] gel and 0.05% NaF); and 3) a combination of the two techniques (APF gel + 0.5% Ca, 0.05% NaF + 0.5% Ca).

**Materials and Methods:**

pH cycling was performed on bovine-tooth specimens alternately immersed in experimental solutions and artificial saliva. After treatment, the microhardness and roughness of the enamel surfaces were measured, and changes in the surface morphology were observed using atomic force microscope images.

**Results:**

Microhardness did not differ statistically significantly between the 2% Ca and mineral water (negative control) groups (2% Ca: 295.34 ± 11.50; mineral water group: 294.76 ± 10.67; p > 0.05). Surface roughness did not differ statistically significantly between the 2% Ca, 0.05% NaF + 0.5% Ca, and mineral water groups (2% Ca: 16.81 ± 7.23; 0.05% NaF + 0.5% Ca: 15.77 ± 2.86; mineral water group: 13.35 ± 5.53; p > 0.05). The surface morphology did not change in the mineral water and 2% Ca groups.

**Conclusion:**

Considering that 2% calcium is a high concentration, adding a low concentration of calcium to fermented milk and applying a low concentration of fluoride daily decreased the reduction of surface microhardness and reduced the increase in surface roughness without causing marked changes in surface morphology. This confirms that combining the two techniques is an effective strategy to prevent dental erosion in-vitro.

Dental erosion is defined as damage to dental hard tissues from the chemical reaction of acids, irrespective of bacterial activity.^50^ Lifestyle changes in recent years, such as increased consumption of acidic drinks, have resulted in an increased prevalence of dental erosion, particularly among children and adolescents, of 30%–59%.^42,48^ In children, the enamel of newly erupted permanent teeth is immature and more vulnerable to damage in an acidic environment;^38^ thus, children should consume acidic drinks with caution. Studies have investigated many techniques to prevent dental erosion caused by the ingestion of acidic drinks. The two most well-known techniques are the addition of calcium to acidic drinks and application of fluoride to the teeth before exposure.

The first method of adding calcium to acidic beverages reduces enamel demineralisation compared with the consumption of acidic beverages without calcium,^18^ with no significant changes in surface microhardness and surface morphology. This demonstrates a protective effect against dental erosion.^24^ Additionally, Barbour and Lussi^4^ reported that acidic products with high calcium and phosphorus contents did not soften dental hard tissues and that addition of calcium was more effective than that of phosphate. Calcium can be safely added to food to prevent dental erosion.^53^ Although the pH and saturation level increase when calcium is added to the erosive solution,^45^ it is important to determine a concentration that is harmless to the human body and prevents dental erosion. Especially a high calcium concentration may increase the risk of developing kidney stones and affect the absorption of other minerals (i.e. zinc, magnesium, and phosphorus).^49^

Many studies have shown that the second method of applying fluoride to teeth before exposure to acidic beverages can prevent dental erosion.^1,7,19,40^ Fluoride is an agent successfully used in the prevention and treatment of tooth demineralisation during dental erosion.^55^ Fluoride is considered an ideal substance for the prevention of dental erosion because it can be easily used in high concentrations in clinical settings and is released slowly.^28^ Further research is required on the various fluoride formulations that aim to prevent dental erosion.

Mature dental enamel cannot spontaneously recover after being exposed to a pathological factor.^37^ Therefore, it is necessary to identify ways to prevent dental erosion. Studies have compared the preventive effect of calcium and fluoride by using both as additives^33^ or as formulations applied directly to tooth surfaces.^34^ However, no study has compared the different methods of adding calcium to acidic beverages and applying fluoride to tooth surfaces. Moreover, many studies have evaluated methods of applying fluoride along with other substances known to prevent dental erosion;^1,22,31^ however, literature on the use of calcium in combination with fluoride is lacking. Therefore, it is necessary to verify whether its ability to prevent dental erosion is improved when calcium is added to acidic beverages, in addition to fluoride application.

Although fermented milk, an acidic beverage, causes dental erosion,^25,29^ *Lactobacillus* in fermented milk inhibits the proliferation of harmful bacteria in the intestines, enhances immunity, decreases morbidity associated with liver cirrhosis, has anti-cancer effects, reduces serum cholesterol, and has dermatological and nutritional benefits.^23^ With increased awareness regarding these benefits, many people consume fermented milk; therefore, it is crucial to minimise dental erosion by identifying preventive measures as opposed to prohibiting its consumption to prevent dental erosion.

Saliva is the primary agent in the oral cavity that prevents dental erosion. As saliva serves as a purifier and buffer while regulating the demineralisation and remineralisation of enamel,^11^ investigations on dental erosion must reproduce the oral environment to consider the possible effects of saliva.

This study aimed to compare the potential to prevent erosion of three techniques, namely: adding calcium to fermented milk, applying fluoride to teeth before exposure to fermented milk, and the combination of both, through pH cycling with tooth specimens alternately immersed in fermented milk and artificial saliva, to identify effective methods to prevent dental erosion.

The null hypothesis of this study was that adding calcium to the fermented milk, applying fluoride before exposing the teeth to the fermented milk, and applying fluoride to the teeth at the same time as adding calcium to the fermented milk cannot prevent dental erosion.

## Materials and Methods

### Materials

#### Experimental solutions

Mineral water (Jeju SamDaSoo, Jeju Special Self-Governing Province Development Corp; Jeju, Republic of Korea) was used as the negative control. With reference to a previous study on the erosion potential of fermented milk currently marketed in the Republic of Korea,^25^ the fermented milk product (Enyo Applecarrot, Maeil Dairies; Seoul, Republic of Korea) with the highest erosion risk was selected as the experimental solution. The fermented milk used in this experiment contained grape juice, apple juice, carrot juice, and 0.5 mg/ml of calcium, with a pH of 3.5. In addition, titratable acidity was measured by the volume needed to reach pH 5.5 and 7.0 by adding 0.05 ml of 1 M NaOH at a time and uniformly stirring. As a result, the titratable acidity was found to be 1.0 and 1.2 ml for pH 5.5 and 7.0, respectively. Moreover, addition of calcium to the fermented milk (2% and 0.5% Ca), topical application of fluoride to the teeth before exposure to the fermented milk (acidulated phosphate fluoride [APF] gel and 0.05% NaF), and a combination of the two techniques (APF gel + 0.5% Ca, 0.05% NaF + 0.5% Ca) were used in this study. The experimental groups are presented in Table 1.

**Table 1:** Experimental groups

Group	n	Treatment
Mineral water (negative control)	12	Mineral water
Fermented milk	12	Fermented milk
2% Ca	12	High-concentration calcium added to fermented milk
0.5% Ca	12	Low-concentration calcium added to fermented milk
APF gel	12	High-concentration fluoride applied once on teeth
APF gel + 0.5% Ca	12	High-concentration fluoride applied once on teeth and low-concentration calcium added to fermented milk
0.05% NaF	12	Low-concentration fluoride applied every day on teeth
0.05% NaF + 0.5% Ca	12	Low-concentration fluoride applied every day on teeth and low-concentration calcium added to fermented milk

APF: acidulated phosphate fluoride.

#### Bovine teeth

For this in vitro study, bovine teeth obtained from a slaughterhouse were adequately washed in running distilled water to remove residues and stored in 70% ethanol until the specimens were produced.

The number of specimens was determined by using G*power 3.1.3 software (Universität Düsseldorf, Düsseldorf, Germany) on previous findings about the prevention of dental erosion caused by fermented milk.^26^ The results showed that 12 specimens for each group had a power of 100%. We prepared 96 specimens without any cracks and having a Vickers hardness number (VHN) of 280–320. These were assigned to eight groups to avoid having statistically significant differences in surface microhardness between the groups.

#### Calcium

Calcium lactate pentahydrate (Junsei Chemical; Tokyo, Japan; molecular formula C_6_H_10_CaO_6_5H_2_O, molecular weight 308.30) was chosen as the erosion preventive agent.

#### Fluoride

A 1.23% acidulated phosphate fluoride (APF) gel (TOPEX Topical A.P.F.* Gel, SultanHealthcare; York, PA, USA) was chosen as the high concentration fluoride for topical application, and 0.05% NaF (Sodium Fluoride, DC Chemical; Seoul, Republic of Korea) solution was chosen as the low concentration fluoride for topical application.

### Methods

#### Specimen preparation

Bovine teeth were placed perpendicular to a 5-mm cylindrical drill and drilled to prepare enamel specimens. A hole was drilled into one side of an acrylic rod to create space for the specimens, which were then placed inside and embedded in acrylic resin. To ensure the presence of smooth surfaces, the specimens were ground with P60, P240, P1, and 200 SiC abrasive paper (CarbiMet, Buehler; Lake Bluff, IL, USA). Then they were polished with a polishing cloth (MicroCloth, Buehler) and aluminum oxide powder (MicroPolish powder, Buehler) using a METASERV 2000 Grinder Polisher (Buehler). After polishing, the specimens were ultrasonicated and washed with distilled water.

The left 1/3 of each prepared specimen was covered with nail varnish (Trendy nails basic original, The Face Shop; Seoul, Republic of Korea) to obtain a surface unexposed to the experimental drink.

#### Initial surface microhardness of specimens

After polishing, the enamel-surface microhardness of the specimens was measured in VHN (Vickers hardness number) using a surface-hardness tester (Fm-7, Future-tech; Tokyo, Japan). The measurements were performed using a Vickers diamond indenter to make 1-mm indentations in the top, bottom, and right-hand edges of the right 2/3 (without nail varnish) of the specimens at 200-gf pressure for 10 s. The indentations were magnified 400X to measure the length and width, and the values were averaged. The average value for the three areas was used as the surface microhardness value.

#### Treatment to prevent erosion

##### Addition of calcium to fermented milk

To prepare the experimental solutions for the 2% and 0.5% calcium groups, 2 g calcium lactate was added to 98 g of fermented milk for the former, and 0.5 g calcium lactate was added to 99.5 g of fermented milk for the latter. The drinks were stirred to completely dissolve the calcium.

##### Fluoride application on specimen surface

The tooth specimens were rinsed in running distilled water and dried with a paper towel. APF gel was applied only once before immersing the specimens in the experimental drink. On each specimen, 1 ml of APF gel was applied using a cotton swab and wiped with gauze after 4 min. The NaF 0.05% solution was applied using a cotton swab for 1 min every night before immersion in the experimental drink, and the specimens were stored under moist conditions until immersion in the experimental drink.

##### Specimen immersion in the experimental solutions

According to the method proposed by Medeiros et al,^34^ immersion in the experimental solutions was set to 5 min, four times a day for 5 days, and the specimens were stored in artificial saliva the rest of the time. Artificial saliva was prepared by adding gastric mucin (0.22%), KCl (0.1114%), KH_2_PO_4_ (0.0738%), NaCl (0.038%), and CaCl_2_·2H_2_O (0.0213%) to distilled water, stirring to completely dissolve them, and adjusting the pH to 7.0 using NaOH. During immersion of the specimens in the experimental drink, the solution was stirred at a speed of 200 rpm such that calcium was not precipitated. While immersed in artificial saliva, the specimens were placed in an incubator at 36.5°C to produce an environment similar to the oral cavity. The experimental drinks and artificial saliva were replaced with fresh solution each time.

#### Assessment of preventive effect on erosion

##### Enamel surface microhardness

Enamel surface microhardness was re-measured after 5 days of pH cycling at the initial measurement site. The difference in VHN (∆VHN) was calculated to indirectly measure the amount of demineralisation caused by dental erosion.

##### Enamel surface roughness

After re-measuring the enamel surface microhardness after 5 days of cycling, the nail varnish on the specimens was carefully removed with acetone (Trendy nails nail-polish remover strawberry, The Face Shop), and the specimens were rinsed with running distilled water to completely remove acetone. In addition, surface roughness (arithmetic average roughness, Ra) was measured in the area to which nail varnish had been applied (control, left 1/3), as well as the area immersed in the experimental drink (treatment, right 2/3) at three points using a contactless optical profiler (Nanosurface 3D optical profiler; 3D OP, NV-E1000, Nanosystem Solutions; Okinawa, Japan), after which the averages were calculated. Further, the difference in the Ra values between the two areas was calculated to assess the change in surface roughness caused by dental erosion.

##### Enamel surface morphology

After measuring surface roughness, representative specimens were chosen from each group, and they were sectioned into 0.5-cm or thinner slices using a specimen cutter (IsoMet low-speed saw, Buehler) and dried at 60°C for 2 days. The surface morphologies of the control (left 1/3) and treatment (right 2/3) areas were observed using an atomic force microscope (AFM, Nanoscope Multimode, Digital Instruments; Woburn, MA, USA) in non-contact mode using a silicon cantilever with a modulus of elasticity of 0.02 to 0.1 Pa, and a length of 450 μm.

### Data Analysis

Twelve specimens were used in each group. As the sample size in each group was less than 30, we performed tests for normality and equal variance. The data were normally distributed and had equal variances; therefore, parametric methods were used for the statistical analyses.

After immersing the specimens in the experimental solutions for 5 days, the ∆VHN was compared within groups using paired t-tests and between groups using one-way ANOVA. The difference in the surface roughness values between the control (left 1/3) and treatment areas (right 2/3) was analysed within groups using paired t-tests and between groups using one-way ANOVA. Tukey’s test was used for post-hoc analysis of all intergroup comparisons. All statistical analyses were performed using SPSS software (version 21.0, IBM; Armonk, NY, USA). The significance level was set to p < 0.05.

The datasets used and/or analysed during the current study are available from the corresponding author upon reasonable request.

## Results

### Changes in Enamel Surface Microhardness

After 5 days of cycling the specimens in the experimental solutions, surface microhardness was statistically significantly reduced in all groups other than the mineral water and 2% Ca groups (p > 0.05, Table 2).

**Table 2:** Differences in enamel surface microhardness after treatment for 5 days

Group	n	Time		Difference**
		Before (day 0)	After (day 5)	
Mineral water (negative control)	12	293.8 ± 9.7	294.8 ± 10.7	- 0.2 ± 7.6^a^
Fermented milk*	12	293.8 ± 9.7	152.2 ± 22.0	- 141.6 ± 18.6^f^
2% Ca	12	293.9 ± 9.8	295.3 ± 11.5	1.4 ± 7.4^a^
0.5% Ca*	12	294.0 ± 10.3	259.4 ± 10.9	- 34.5 ± 13.5^c^
APF gel*	12	294.1 ± 11.3	190.1 ± 25.0	- 104.0 ± 21.2^e^
APF gel + 0.5% Ca*	12	294.3 ± 10.5	258.0 ± 12.8	- 36.3 ± 11.2^c^
0.05% NaF*	12	294.6 ± 12.4	230.2 ± 18.4	- 64.4 ± 17.2^d^
0.05% NaF + 0.5% Ca*	12	294.8 ± 12.7	276.0 ± 10.7	- 18.7 ± 9.9^b^

Values are given as mean ± SD; unit of surface microhardness: VHN. *p < 0.05, paired t-test. **p < 0.05, one-way ANOVA. Same superscript letters indicate no statistically significant difference by Tukey’s test at α = 0.05. APF: acidulated phosphate fluoride.

∆VHN statistically significantly differed among the groups, with a range of -141.64 to 1.41 VHN (p < 0.05, Table 2). ∆VHN did not statistically significantly differ between the 2% Ca and mineral water groups (p > 0.05). However, ∆VHN in the 0.5% Ca, APF gel, APF gel+ 0.5% Ca, 0.05% NaF, and 0.05% NaF + 0.5% Ca groups were statistically significantly different from those in the mineral water group and fermented milk group (p < 0.05).

### Changes in Enamel Surface Roughness

After 5 days of cycling the specimens in the experimental solutions, surface roughness was statistically significantly reduced in all groups other than the mineral water and 2% Ca groups (p > 0.05, Table 3).

**Table 3:** Differences in enamel surface roughness (µm) after treatment for 5 days

Group	n	Ra		Difference**
		Control	Treatment	
Mineral water (negative control)	12	15.0 ± 4.1	13.4 ± 5.5	- 1.6 ± 5.4^a^
Fermented milk*	12	16.5 ± 3.5	153.1 ± 39.3	136.6 ± 40.4^c^
2% Ca	12	18.3 ± 7.7	16.8 ± 7.2	- 1.5 ± 6.0^a^
0.5% Ca*	12	13.8 ± 4.0	61.8 ± 15.3	48.0 ± 16.8^b^
APF gel*	12	14.2 ± 3.2	141.9 ± 44.1	127.7 ± 45.7^c^
APF gel + 0.5% Ca*	12	15.3 ± 3.3	57.1 ± 15.7	41.7 ± 15.5^b^
0.05% NaF*	12	14.6 ± 4.0	70.8 ± 24.6	56.2 ± 22.7^b^
0.05% NaF + 0.5% Ca *	12	14.0 ± 2.1	15.8 ± 2.9	1.8 ± 3.1^a^

Values are given as mean ± SD; surface roughness given as Ra (µm). *p < 0.05, paired t-test. **p < 0.05, one-way ANOVA. Same superscript letters indicate no significant difference in Tukey’s test at α = 0.05. APF: acidulated phosphate fluoride.

Furthermore, the change in surface roughness statistically significantly differed among the groups, with a range of -1.63 to 136.56 Ra (p < 0.05, Table 3). The change in surface roughness in the 2% Ca and 0.05% NaF + 0.5% Ca groups was not statistically significantly different from that in the mineral water group (p > 0.05). The change in surface roughness in the 0.05% Ca, APF gel + 0.5% Ca, and 0.05% NaF groups statistically significantly differed from those in the mineral water and fermented milk groups (p < 0.05), while it did not differ statistically significantly in the APF gel group compared with that in the fermented milk group (p > 0.05).

### Changes in Enamel Surface Morphology

**Fig 1:**
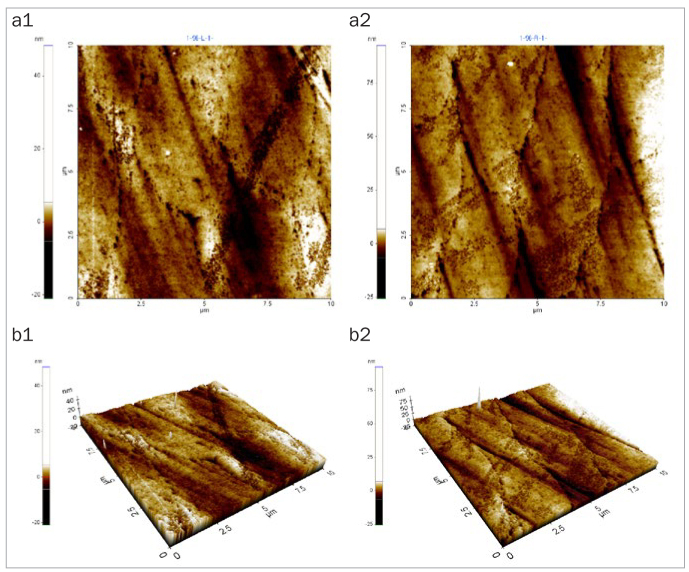
AFM image of the mineral water group. There was no marked change in surface morphology. a1: 2D image of the enamel surface before treatment; a2: 2D image of the enamel surface after treatment; b1: 3D image of the enamel surface before treatment; b2: 3D image of the enamel surface after treatment. AFM: atomic force microscope; 2D: two-dimensional; 3D: three-dimensional.

**Fig 2:**
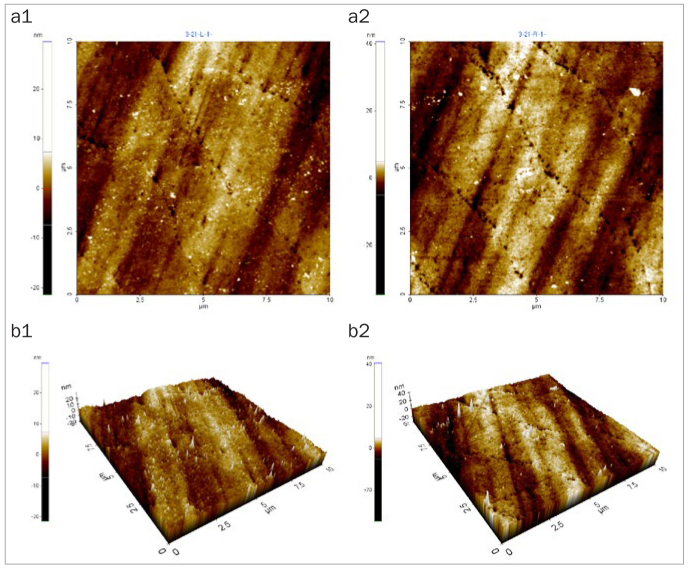
AFM image of the fermented milk group shows dark, round holes that resemble craters. a1: 2D image of the enamel surface before treatment; a2: 2D image of the enamel surface after treatment; b1: 3D image of the enamel surface before treatment; b2: 3D image of enamel surface after treatment. AFM: atomic force microscope; 2D: two-dimensional; 3D: three-dimensional.

**Fig 3:**
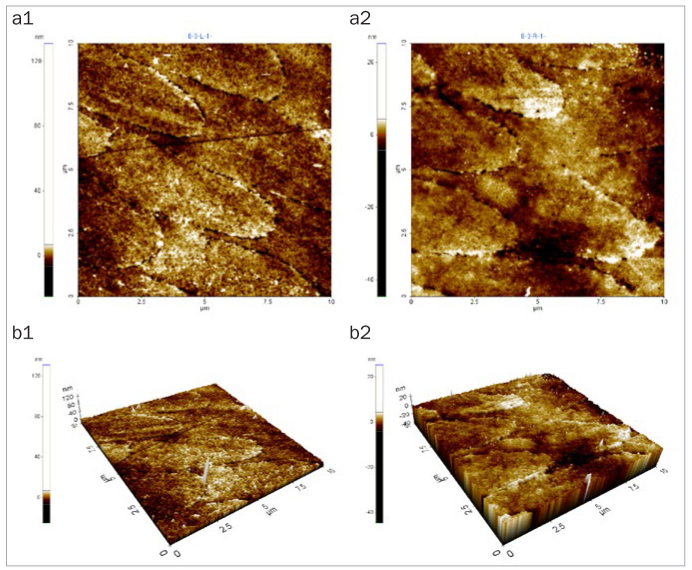
AFM image of the 2% Ca group. No marked change in surface morphology was observed. a1: 2D image of the enamel surface before treatment; a2: 2D image of the enamel surface after treatment; b1: 3D image of the enamel surface before treatment; b2: 3D image of enamel surface after treatment. AFM: atomic force microscope; 2D: two-dimensional; 3D: three-dimensional.

**Fig 4:**
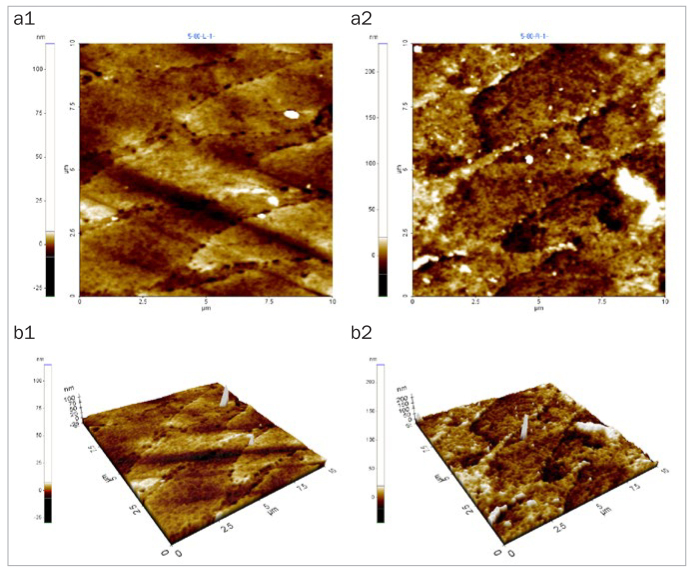
AFM image of the 0.5% Ca group. Mild surface damage morphology was observed. a1: 2D image of the enamel surface before treatment; a2: 2D image of the enamel surface after treatment; b1: 3D image of the enamel surface before treatment; b2: 3D image of enamel surface after treatment. AFM: atomic force microscope; 2D: two-dimensional; 3D: three-dimensional.

**Fig 5:**
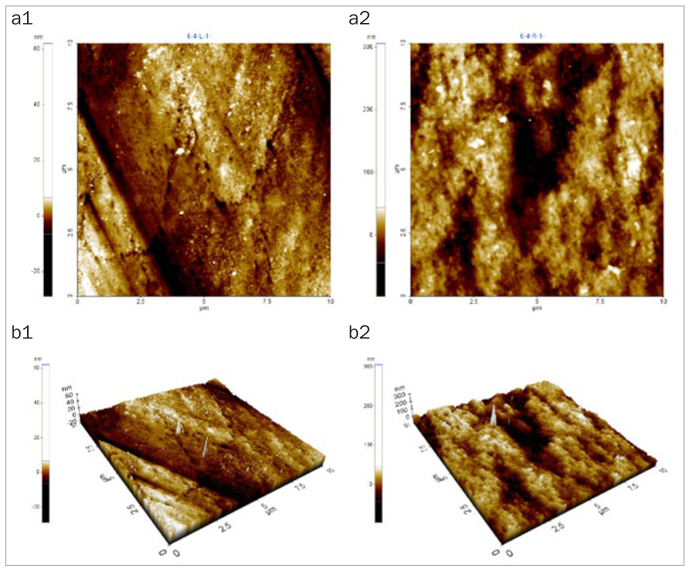
AFM image of the APF gel group. Irregular and unique arrangements of particles at different heights were observed. a1: 2D image of the enamel surface before treatment; a2: 2D image of the enamel surface after treatment; b1: 3D image of the enamel surface before treatment; b2: 3D image of enamel surface after treatment. AFM: atomic force microscope; 2D: two-dimensional; 3D: three-dimensional.

**Fig 6:**
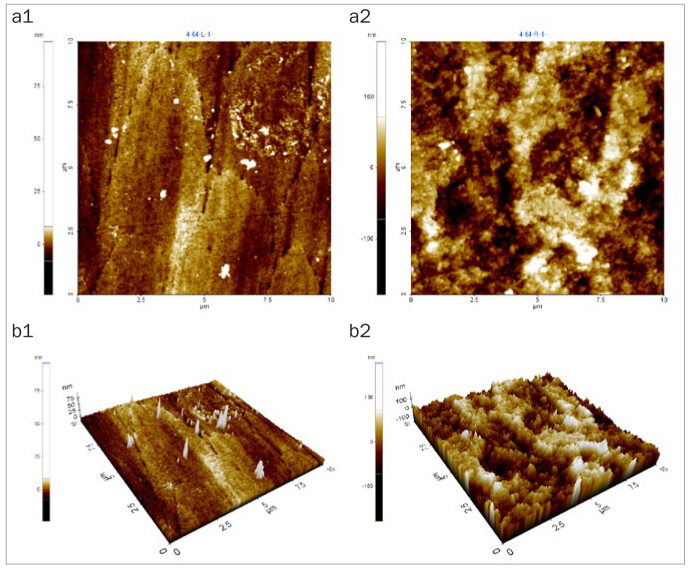
AFM image of the APF gel + 0.5% Ca group. A greater extent of damage was observed compared with that in the 0.5% Ca group. a1: 2D image of the enamel surface before treatment; a2: 2D image of the enamel surface after treatment; b1: 3D image of the enamel surface before treatment; b2: 3D image of enamel surface after treatment. AFM: atomic force microscope; 2D: two-dimensional; 3D: three-dimensional.

**Fig 7:**
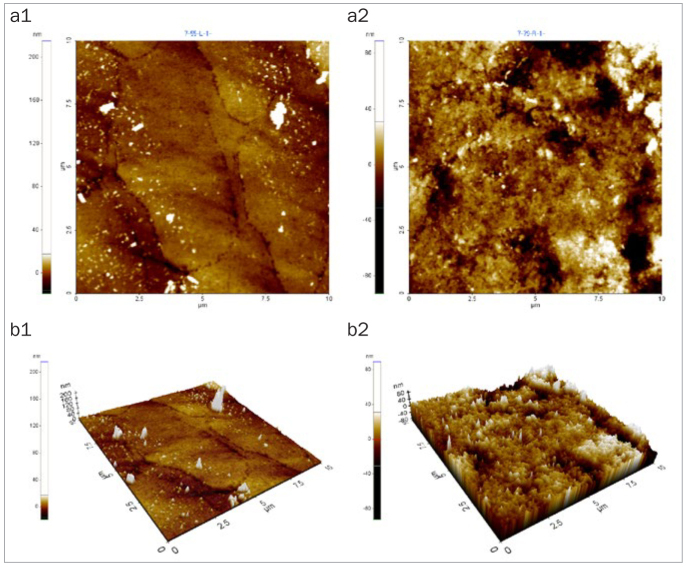
AFM image of the 0.05% NaF group. A surface damage with an irregular arrangement was observed. a1: 2D image of the enamel surface before treatment; a2: 2D image of the enamel surface after treatment; b1: 3D image of the enamel surface before treatment; b2: 3D image of enamel surface after treatment. AFM: atomic force microscope; 2D: two-dimensional; 3D: three-dimensional.

**Fig 8:**
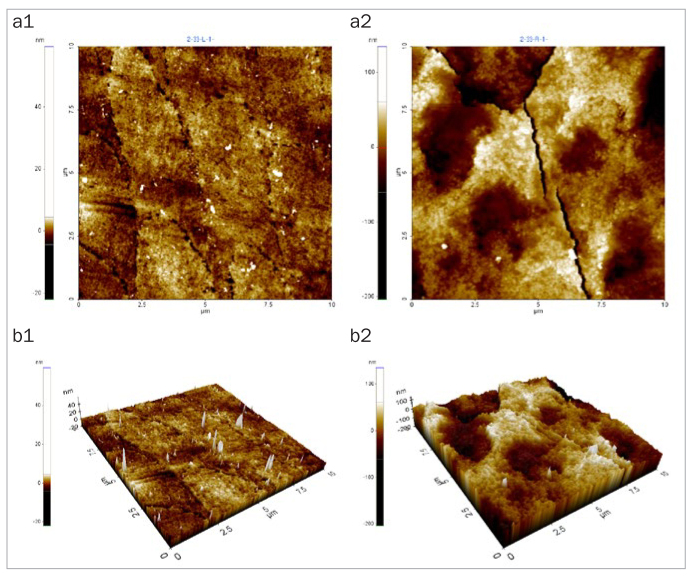
AFM image of the 0.05% NaF+ 0.5% Ca group. There was no marked change in the surface morphology on 2D images, but mild surface damage was observed on 3D images. AFM: atomic force microscope; 2D: two-dimensional; 3D: three-dimensional.

After 5 days of cycling the specimens in the experimental solutions, there was no marked change in the surface morphology in the mineral water group (Fig 1), while the fermented milk group showed the greatest damage to surfaces due to dental erosion, with dark round holes that resembled craters (Fig 2). The 2% Ca group also failed to show a statistically significant change in the surface morphology, as in the mineral water group (Fig 3). However, the 0.5% Ca group showed uneven surfaces with dark spots throughout the specimens, indicating damage (Fig 4). The APF group showed irregular and unique patterns, as well as more surface damage than that observed in the APF gel + 0.5% Ca group (Fig 5). The APF gel + 0.5% Ca group was more damaged than the 0.5% Ca group, although 0.5% Ca was added to the fermented milk and APF gel was applied to the specimen (Fig 6). Nevertheless, our findings confirmed that the surface morphology was altered less when 0.5% Ca was added to the fermented milk than when only APF gel was used for treatment. In addition, although the surface alteration depth in the 0.05% NaF group was not as high as that in the APF gel group, surface damage could be observed with an irregular arrangement (Fig 7). Finally, while there was no marked change in the surface morphology on the two-dimensional (2D) images for the 0.05% NaF + 0.5% Ca group, mild surface damage was observed on the three-dimensional (3D) images, although the surface was relatively even (Fig 8).

## Discussion

In this study, the addition of calcium to fermented milk and application of fluoride to the teeth before exposure to fermented milk were chosen as methods to prevent dental erosion caused by consuming fermented milk. Franklin et al^16^ found that fruit juice with added calcium had improved pH and buffering capacity, and enamel mineral loss was reduced in human teeth treated with it compared with fruit juice without calcium. In an in-situ study using bovine teeth, Scaramucci et al^44^ observed that adding a small amount of calcium to orange juice reduced its erosive potential. Calcium not only helps reduce dental erosion but can also strengthen human bone tissues. Therefore, calcium-enhanced drinks are becoming more popular in the market.^47^ However, due to safety considerations, the amount of calcium added to food products is limited; moreover, calcium alters the flavour of drinks.^5^ Furthermore, it increases the risk of kidney stones and hinders the absorption of other minerals, such as zinc, magnesium, and phosphorus.^49^ It is necessary to explore methods of providing an effective yet safe dose of calcium to prevent dental erosion. In this study, with reference to Kim et al,^26^ 0.5% was chosen as the low concentration of calcium, at which surface microhardness began to statistically significantly differ, and 2% was chosen as the high concentration of calcium, at which no statistically significant differences were observed compared with that of mineral water.

In addition to adding calcium to acidic drinks, the application of fluoride to teeth has been documented in many studies as preventing dental erosion.^1,7,19,40^ Fluoride combines with hydroxyapatite in the enamel to form fluorapatite, which makes the crystals more compact,^2^ thereby increasing the resistance to demineralisation caused by acidic drinks. Furthermore, calcium fluoride (CaF_2_) formed on tooth surfaces upon application of high-concentration fluoride serves as a mechanical barrier that prevents contact with acid and also serves as a mineral reserve.^13^ De Carvalho Filho et al^12^ applied fluoride varnish and gel to bovine teeth before treating them with acidic drinks and observed them using an energy-dispersive x-ray fluorescence device. They found that although both fluoride varnish and gel protected the teeth, fluoride gel provided the best protection against exogenous dental erosion at a lower cost. Hence, in this study, we selected APF gel as a high-concentration topical fluoride. In addition, although patients are recommended to apply fluoride gel every 6 months in clinical practice, we only applied fluoride once before the treatment. Furthermore, to evaluate the effect of daily mouthrinsing with fluoride, we chose 0.05% NaF as a low-concentration fluoride and applied it to the specimen for 1 min every evening before treatment with the experimental drink.

First, the changes in enamel surface microhardness were assessed using a surface hardness tester. This is a general method for quantifying the impact of dental erosion; changes in the surface layer are measured based on the indentation left by a diamond-shaped indenter.^3^ Because this method has been reported to be appropriate for observing minor changes in surface microhardness from erosion,^32^ it was chosen to indirectly measure and assess the degree of demineralisation caused by dental erosion. After immersing the specimens in fermented milk and artificial saliva for 5 days, only the 2% Ca group showed no statistically significant changes in microhardness compared with baseline, indicating that this method has the highest potential for erosion prevention. The five other experimental groups did not completely prevent dental erosion; however, they showed differences in the reduction of microhardness compared with the fermented milk group, although the degree varied. The APF gel group showed the highest change in surface microhardness, indicating that it had the lowest erosion prevention compared with the other conditions tested. These results suggested that daily application of a low-concentration fluoride would lead to better prevention of erosion than a single application of a high-concentration fluoride when fermented milk is consumed over a long period. Although the 2% Ca group showed the highest erosion prevention, 2 g of calcium was added to the solution; thus, the upper limit of calcium intake should be considered when applying this method in-vivo. According to The Korean Nutrition Society,^35^ the recommended daily upper limit for calcium is 2.5 g for infants and children aged 1–8 years and 3.0 g for children and adolescents aged 9–18 years; hence, 2% calcium is close to the upper limit for the younger age groups. Adding a low concentration of calcium to fermented milk and applying a low-concentration fluoride on teeth every day could be a good alternative with excellent erosion prevention.

The second method used to assess erosion prevention was to measure enamel surface roughness. The differences in the surface height of uneven enamel were presented as Ra using a contactless 3D optical profiler. As this method is contactless, it overcomes the shortcomings of devices that require direct contact. Interestingly, a previous study reported consistently satisfactory results over the past few years.^17^ In our study, only the mineral water and 2% Ca groups showed no changes in surface roughness. Furthermore, the 0.05% NaF% + 0.5% Ca group showed a statistically significant increase in surface roughness; however, when compared with other groups, it was not significantly different compared with that of the mineral water and 2% Ca groups. In addition, the APF gel group showed the highest surface roughness among the treatment groups, with no significant difference from the fermented milk group. Increased enamel surface roughness not only heightens the vulnerability to microbial attachment and biofilm deposition, but it may also cause irreversible pooling of microbial cells.^41^ Therefore, greater surface roughness caused by dental erosion is highly relevant to oral health.

The third method of assessment of erosion prevention was to observe changes in the enamel surface morphology using AFM. In this study, we used non-contact mode, leaving space between the tip and the specimens in order to minimise damage. In dentistry, AFM is used to observe the surfaces of teeth, restoration materials,^36^ implants,^39^ and orthodontic devices,^10^ as well as research on dental erosion prevention, such as in our study.^43^ When observed using an AFM, only the 2% Ca group among the six treatment groups showed no changes in surface morphology. Although the 0.05% NaF + 0.5% Ca group showed no changes in surface morphology in the 2D images, mild surface damage was evident in the 3D images. The APF gel group showed the greatest surface damage, which was consistent with the surface microhardness and roughness results. The remaining treatment groups also showed varying levels of surface damage.

Surface roughness and surface morphology were assessed by comparing the area of a specimen that was covered with nail varnish to prevent exposure to the experimental solution vs the area of the same specimen that had been exposed to the experimental solution. We examined whether the nail varnish and acetone used in this experiment affected the specimens. Hence, as a preliminary analysis, the specimen surface was observed using a microscope before applying nail varnish, and hardness was measured using a surface hardness tester. The surface morphology and hardness were re-examined after applying nail varnish and removing it with acetone. No marked changes were observed before and after applying nail varnish; therefore, we decided to use nail varnish for the experiment.

In this study, we assessed changes in surface microhardness, surface roughness, and surface morphology to identify methods effective in preventing dental erosion caused by fermented milk. When treated with fermented milk for 5 days, the APF gel + 0.5% Ca and NaF 0.05% + 0.5% Ca groups showed better inhibition of demineralisation than the APF gel and 0.05% NaF groups, respectively, suggesting that adding calcium increases the erosion-preventive potential. Kim et al^26^ observed that the pH of fermented milk increases with increasing calcium content, known to be attributable to the chelation of calcium by acidic ions in fermented milk, thus reducing the content of active acid.^16^ Furthermore, as it has been reported previously that the possibility of erosion within the first few minutes after exposure to a drink is completely dependent on the pH of the drink,^20^ we speculate that the erosion-preventive potential increased in the groups with added calcium due to elevated pH. Lodi et al^30^ reported that some characteristics, such as low pH and high buffering capacity of fermented beverages, may promote demineralisation of the dental enamel. However, fluoride, calcium, and phosphorus may reduce the dissolution of dental enamel by acidic substances. Interest in certain foods that promote health or are known as functional dairy products is increasing.^15^ Fermented milk is a dairy product that falls under this category.^14^ Therefore, in this study, we aimed to investigate the effect of fermented milk consumption on the tooth surface when ingested for 5 days, which is a relatively long period of time. It was expected that tooth erosion due to enamel demineralisation would increase as time passed, even if only superficial mineral loss occurred in the initial stage.

In addition, it was found that a single application of high-concentration fluoride, such as APF gel, had the lowest erosion-preventive potential. Previous studies by Calvo et al^6^ and Villena et al^52^ observed the inhibitory effect of enamel demineralisation of APF gel and reported that demineralisation was reduced due to calcium fluoride and fluoroapatite formation. In the aforementioned studies, direct acid contact was not made because the palatal appliances containing enamel blocks were immersed in sucrose. In this study, as fermented milk was directly exposed to the specimen, fluoride gel had a low preventive effect on dental erosion. Several studies, including the present, have confirmed that application of fluoride at a low concentration is more effective in preventing dental erosion than high concentrations of fluoride. Carvalho et al^8^ reported that applying a high-concentration fluoride varnish on teeth before immersing the teeth in a cola beverage did not prevent dental erosion, and hypothesised that the calcium fluoride formed before deposition of fluorapatite (after fluoride application) was easily dissolved in acidic drinks.^27^ Furthermore, we observed that the addition of low-concentration calcium with a single application of high-concentration fluoride had a lower erosion-preventive potential than the addition of low concentrations of calcium to fermented milk with a daily application of low-concentration fluoride. In a study comparing the prevention of enamel erosion between commercial fluoride varnish and fluoride varnish containing calcium glycerophosphate, Carvalho et al^9^ found that fluoride varnish containing calcium glycerophosphate did not improve fluoride binding on the enamel compared with commercial fluoride varnish. Thus, those authors concluded that it did not improve the protective effects against enamel erosion; especially the erosion-preventive effects of fluoride varnish were not statistically significantly better after adding calcium. In contrast, Turssi et al^51^ reported that rinsing teeth with calcium lactate before rinsing with a low-concentration fluoride statistically significantly reduced enamel surface loss compared with rinsing teeth with fluoride alone. In this study, we also confirmed that daily application of low concentrations of fluoride with the addition of low concentrations of calcium in fermented milk is effective in preventing dental erosion. Considering that fluoride formulations previously tested for erosion prevention were highly concentrated, such as fluoride varnish and fluoride gel, it is necessary to investigate the effects of continuous application of low concentrations of fluoride.

In this study, we assessed calcium and fluoride as erosion-preventive agents using sound enamel specimens, and the results showed that calcium had better overall potential than fluoride. However, fluoride has a high acid resistance and remineralisation effect; therefore, fluoride may show better results if assessed on enamel specimens with early caries. After remineralising teeth using a solution containing low-concentration calcium, Silverstone^46^ reported that the width of the lesion decreased by 22%; however, after the addition a low concentration of fluoride (1 ppm) to the low-concentration calcium solution, the width of the lesion decreased by 72%. Therefore, additional studies on incipient or progressive carious lesions are needed. Moreover, this study was conducted in a laboratory using artificial saliva. Although using human saliva is considered the best method to reproduce the oral environment, inter-individual variations may exist as saliva from several people must be used. Furthermore, artificial saliva was selected because a previous study by Jeong et al^21^ reported that the remineralisation effect of artificial saliva is similar to that of human saliva, and it was always used under the same conditions. Nevertheless, the characteristics of human saliva cannot be reproduced perfectly, so that differences between in-vitro and in-vivo conditions may exist due to the presence of various substances other than saliva in the oral environment. Therefore, more accurate results may be obtained if the in-vivo model could be evaluated using an intraoral device in the future.

If the tooth surface is lost due to demineralisation by dental erosion, healthy inner enamel or dentin not affected by acidic drinks may be exposed. This may cause microhardness results that could be underestimated even if the surface is actually damaged by dental erosion; therefore, we also measured the surface roughness and observed surface morphology. However, these methods only assess tooth surfaces, and it may be necessary for erosion-related studies to assess changes according to enamel depth. Thus, future studies should consider measuring the thickness of enamel or mineral content according to enamel depth.

Consumption of acidic drinks has increased worldwide over the last few decades, making dental erosion a subject of continued interest. Thus, it is important to assess the risk of, prevent, and manage dental erosion. To prevent dental erosion, Zero and Lussi^54^ proposed means such as decreasing the duration and frequency of acid contact in the mouth by not holding or rinsing acidic drinks in the mouth, and consuming modified acidic drinks that can eliminate or reduce the possibility of dental erosion. As shown in our results, applying fluoride to teeth in addition to consuming modified acidic drinks containing calcium would prevent dental erosion more effectively.

The findings of this study may help increase the awareness of the risk of dental erosion caused by exposure to fermented milk, if no preventive treatments are undertaken, and further may help children and their parents recognise the need for prevention. We have also proposed effective preventive methods and appropriate alternatives for people at high risk of dental erosion.

## Conclusion

Effective means of preventing dental erosion caused by fermented milk were examined using sound enamel specimens. Adding 2% calcium to fermented milk before immersion did not lead to statistically significant changes in surface microhardness, surface roughness, or surface morphology compared with the baseline. However, considering that 2% calcium is a high concentration, adding a low concentration of calcium to fermented milk and applying a low concentration of fluoride daily decreased the reduction in surface microhardness and reduced the increase of surface roughness without causing marked changes in surface morphology. These findings confirmed that combining both techniques is an effective erosion-preventive strategy and provided an effective means in-vitro to prevent dental erosion in individuals at high risk of dental erosion. Considering our findings, the null hypothesis of the study was rejected.
